# The seven deadly sins writers of academic papers should avoid

**DOI:** 10.1080/13814788.2017.1384809

**Published:** 2017-10-18

**Authors:** Igor Švab

**Affiliations:** Medical Faculty, Department of Family Medicine, University of Ljubljana, Ljubljana, Slovenia


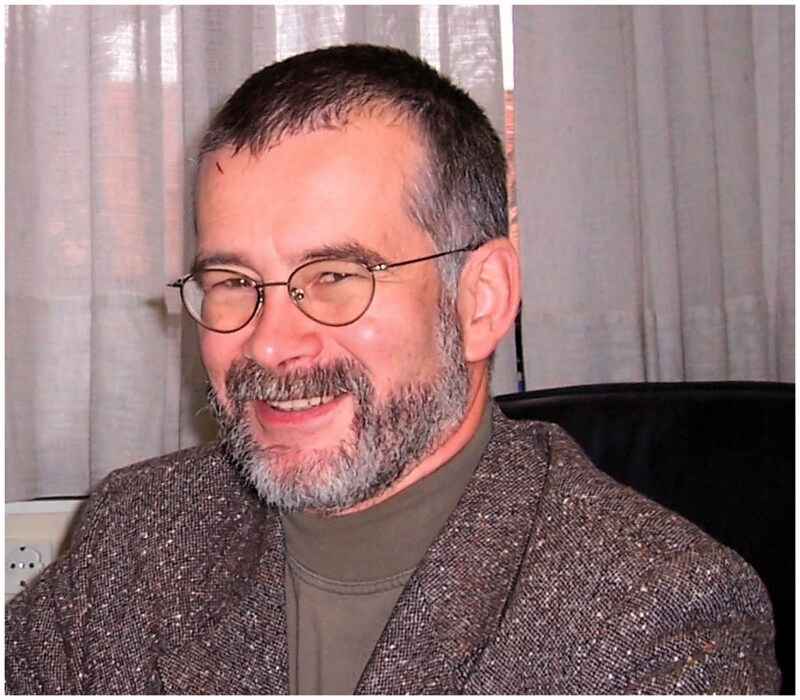


Being an editor of a scientific journal is a challenging job. The editor’s task is to select articles regarding quality and interest. This is not always easy: we accept approximately 25% of manuscripts we receive. The obvious selection criterion is the quality of the manuscript. However, sometimes a paper suffers from other issues and these problems are not related to the scientific quality of the paper but to the proper conduct of the authors. Sometimes they are suspected early in the editorial process but sometimes even after the paper has been published.

## Seven sins and how they are dealt with

What are the most frequent and grave errors we as editors encounter?

### Technical sloppiness

Technical sloppiness is not a problem of misconduct but is included here because it is an important cause for a paper to be rejected early. Quite often papers we receive are not prepared according to the technical standards of our journal. Sometimes a statement on the medical research ethics committee is missing. Sometimes authors have not been careful enough in reading the instructions for authors. However in other cases, we notice that the paper has obviously been sent elsewhere, rejected and then simply resent to our journal. In that case, the article is sent back to the authors with a request that the paper should be prepared correctly. This kind of error does not improve the chance of the paper to be published.

### Conflict of interest

Editors tend to become suspicious if the cover letter does not mention conflicts of interest. If there is any potential conflict, this should be mentioned not only in the cover letter but also in the main text of the manuscript since reviewers need to be informed about this issue as well. Most studies are sponsored and sponsoring may affect the objectivity of the paper. Not mentioning conflict of interest, especially in sponsored research is bad practice. Not only ‘material’ conflicts of interest should be mentioned, but also ‘immaterial’ interests—like being co-author of a clinical guideline, or being a member of a special interest group need to be acknowledged. In particular, the authors of a ‘background paper’ or ‘narrative review’ should be transparent about the perspective from which they write a paper.

### Plagiarism

It has become common practice for most journals (ours included) to use computer programmes that check databases for texts to screen for possible plagiarism. Real plagiarism, where substantial parts of an article written by someone else are being reprinted and submitted as an original paper from another author is rather rare. Often, an expert reviewer informs the editor that parts of the manuscript seem to be copied from a text they know. Needless to say, such a paper will be rejected and the authors will be asked for an explanation. Depending on their answer, more steps that are serious may follow, like informing superiors or the dean of a university.

Self-plagiarism is more common and sometimes necessary: it is perfectly logical to have similar paragraphs in the method sections of papers reporting on the same dataset. However, sometimes we come across a paper that has been originally published in a non-English journal and is then submitted in English. For an editor, it is hard to judge whether this is done on purpose—and the authors hope that this will not be spotted—or whether it is ignorance of the authors. If the authors did not mention the other publication in their cover letter, we will not negotiate about a possible allowed ‘duplicate publication.’

Other commonly encountered issues are partially overlapping and ‘salami’ publications. Then, an editorial decision is less straightforward. If a prospective study has outcomes after three, six and 12 months, it will be hard to defend that this situation warrants three separate publications. The last paper of such a series will be rejected if there is too much repetition of the text, especially if previously published results are repeated (i.e., almost identical tables across various articles).

There may be legitimate reasons for the authors to produce several articles from the same study. Sometimes the methodology of the study is so complex that describing it in detail may require too much space, and a separate methodological paper is published. An overview of the literature, if done systematically, may be a separate paper or a systematic review. International studies may make it possible to produce ‘national’ sub-papers which would be interesting for a national journal. Some ‘self-plagiarism’ cannot be avoided, but authors should try to prevent too much overlap with their work.

It is good practice that authors are transparent about previous, similar, or associated publications (in any language). In all these cases, authors should mention their previous publications on a similar topic in the cover letter, and they should explain the benefit of the current manuscript.

### Double submission

Sometimes, authors decide to submit the same paper—or a similar one, e.g., a more comprehensive version—to several journals at the same time. Some authors, usually the inexperienced ones, not well supervised by a senior are not aware that double submission is not allowed. It is clear that this is explicitly forbidden according to international guidelines on publication ethics [1]. If it happens, it is considered to be a grave mistake, i.e., the editor of the other journal will be notified, as will the superiors of the authors, e.g., the dean of a university.

### Self-citation

It is logical that authors quote their previous work in a new manuscript. However, there must be a balance between own work and work of others on the same topic. It is bad practice to list only the author’s previous work in the references, giving the impression that the author is the only researcher on the topic. The authors must be critical enough and acknowledge the work of other research groups working on the same topic by quoting their studies.

### Data manipulation

Sometimes the data are fabricated, modified or changed. Sometimes authors decide to report only the part of their results that support their theory, hypothesis or interest, and neglect to mention the other results. These are serious cases of misconduct [1]. It is also very difficult for editors to discover that kind of misconduct—although expert reviewers may raise suspicions. It is often recognized after the study has been repeated [2]. In that case, the editor officially retracts the article, sometimes with an accompanying explanation. This causes serious discrediting of the author, which critically affects his career.

Sometimes serious misconduct occurs it is possible to inform the authorities. This is done extremely rarely, but this year we had the first case when it was discussed at the editorial board meeting.

### Wrong and honorary authorship, ghost-writing, guest-writing and gift-writing

In principle, the rules of authorship are clear. The authors are people who have substantially participated in various stages of the study. Less substantial contributions should be acknowledged as such and should not be awarded a ‘gift,’ ‘guest’ or ‘honorary’ authorship. Ghostwriting (when somebody else writes the paper) is forbidden. A professional writer, often paid by the commercial sponsor of a study or ‘background paper,’ may do this.

To qualify as an author, the authors should:

- Make substantial contributions to the conception or design of the work; or the acquisition, analysis, or interpretation of data for the work; and
- Draft the work or revise it critically for important intellectual content; and
- Make final approval of the version to be published; and
- Agree to be accountable for all aspects of the work in ensuring that questions related to the accuracy or integrity of any part of the work are appropriately investigated and resolved [3].

It is hard for the editorial office to find out if an author is real. Google and PubMed may reveal a lot of information, which either may increase or decrease suspicion that authorship may be an issue. It is important for co-authors of a paper to be aware that they have a shared responsibility for their collective authorship.

Almost all conflicts of authorship are made known only after the paper has been published and create conflicts between the authors that can easily be avoided. Many friendships are broken over the issue of authorship.

## What can authors do?

Authors are expected to know what is considered ‘good practice’ in academic writing. We suggest you have a good look at the websites of the International Committee of Medical Journal Editors [3] and the Committee on Publication Ethics [1]. It is interesting to see that they are sometimes unaware that double submission is considered misconduct. Naïve beginners make this mistake by accident. It is the role of senior researchers, their tutors and supervisors to ensure that they are aware of that.

Here is a simple checklist that every author could use before submitting a paper to a journal to avoid problems and allegations of possible misconduct:

1. Be clear about who you are. Try to make yourself known through ORCID, a university web page, LinkedIn or other sources that are well known. This makes the work of editors much easier. Check the affiliations you provide in the electronic manuscript management system and on the ‘author page’. Use official stationery or logos in your cover letter or email address.
2. Adhere to the instructions for authors. Did you formally prepare your manuscript according to the requirements of the journal?
3. Do your co-authors fulfil the criteria for authorship? Is a long list of authors explained in the cover letter? Have they all agreed to be a co-author? Is that stated in the cover letter?
4. Does the cover letter include bibliographic details of already published articles—in any language—by the same author group? Do you discuss the benefit of the current manuscript if it looks similar to an article you have published already?
5. Did you not simultaneously submit the same manuscript to another journal? Did you include a statement on that in the cover letter?
6. Did you mention possible conflicts of interest in the cover letter as well as the text of the manuscript?
7. Are details of medical ethics committee approval provided?

## Conclusions

Editors have to be suspicious and look for clues how to find cases of misconduct. Even with the utmost care, it is always possible to cheat and misconduct will never be eradicated. However, the main responsibility lies with the author, who should make it clear that professionalism, research and publication ethics are more important than scores and factors based on publication of controversial articles.

Very little is clear-cut in terms of finding and investigating misconduct, including the boundaries of responsibilities and how far to take an investigation. In the *European Journal of General Practice,* we are dealing with these problems in a fair but strict way. After all, we are family doctors.
